# Spiders (Arachnida: Araneae) in the semideciduous Atlantic Forest: An ecological and morphological trait dataset for functional studies

**DOI:** 10.3897/BDJ.8.e49889

**Published:** 2020-03-12

**Authors:** Ana Munévar, Pedro Cardoso, Yolanda M.G. Piñanez Espejo, Gustavo Andres Zurita

**Affiliations:** 1 Instituto de Biología Subtropical (UNAM-CONICET), Puerto Iguazú, Argentina Instituto de Biología Subtropical (UNAM-CONICET) Puerto Iguazú Argentina; 2 Laboratory for Integrative Biodiversity Research (LIBRe), Finnish Museum of Natural History, University of Helsinki, Helsinki, Finland Laboratory for Integrative Biodiversity Research (LIBRe), Finnish Museum of Natural History, University of Helsinki Helsinki Finland; 3 Instituto de Biología Subtropical, Universidad Nacional de Misiones–CONICET, Puerto Iguazú, Misiones, Argentina. Facultad de Ciencias Forestales, Universidad Nacional de Misiones, Misiones, Argentina Instituto de Biología Subtropical, Universidad Nacional de Misiones–CONICET, Puerto Iguazú, Misiones, Argentina. Facultad de Ciencias Forestales, Universidad Nacional de Misiones Misiones Argentina

**Keywords:** Araneae, functional, traits, subtropical, forest, pine, plantations

## Abstract

**Background:**

The semideciduous Atlantic Forest is one of the most diverse ecosystems in the world with a great diversity of spiders. Most spider-related studies in this ecosystem focused on species richness and composition; however, little is known about their trait diversity (including morphological, ecological and/or physiological traits). Two main datasets were compiled to generate a complete record of spider traits for this ecosystem.

**New information:**

Here, we present two datasets about 259 species of spiders from the semideciduous Atlantic Forest of Argentina. The trait data set compiled information of morphological and ecological traits such as body size, femur length, ocular distance, foraging strategy, prey range, circadian activity and stratum preference; traits were assessed by species considering sexual dimorphism. The second dataset included information about phenology (season when spiders were collected), number of individuals assessed by species and presence/absence of spiders in the different sample sites. This dataset has high potential to help researchers in recording the state of a component of biodiversity (functional) and contributes with the study of ecosystem services and species conservation.

## Introduction

The Atlantic Forest of Argentina, Brazil and Paraguay is one of the most diverse ecosystems in the world; this biome hosts about 7% of the global known species richness and shows high levels of endemism (Oliveira-Filho and Fontes 2000, Eisenlohr et al. 2015). However, 90% of the Atlantic Forest has been replaced by intensive productive systems such as crops, livestock and tree plantations (Di Bitteti et al. 2003). The southern portion of the Atlantic Forest, located in the province of Misiones, Argentina and known as the semideciduous Atlantic Forest, preserves the largest continuous remnants of this ecoregion (Galindo-Leal and Câmara 2005).

Previous studies in the semideciduous Atlantic Forest have reported 550 species of spiders in Argentina (Rubio 2016, Rubio et al. 2018), 448 in Santa Catarina and 183 in Estado de Paraná, Brazil (Gonçalves da Rosa et al. 2019, Brito Pitilin et al. 2019). In contrast to the taxonomic approach, which focuses on species identity, functional diversity is a complementary approach that assumes species are not equal in the context of ecosystem functioning and their response to disturbances (Legras et al. 2018). However, functional studies require that morphological, ecological, physiological and behavioural traits of species are described (Violle et al. 2007).

In the Atlantic Rain Forest (northeast of Brazil), Gonçalves-Souza et al. (2014) described traits for 176 species of spiders; authors compiled four morphological (adhesive structures, eye arrangement/tapetum, body size and compression) and three ecological traits (sheltering behaviour, foraging period and mode) of spiders. In the semideciduous Atlantic forest, Raub et al. (2015) assessed functional diversity of secondary forests based on ecological traits of 220 species/morphospecies of spiders. Despite the species richness included in both studies, neither trait matrix (traits by species) nor details about number of individuals/species and sex considered were available.

Here, we present one of the most complete datasets of morphological and ecological traits for spiders inhabiting both native forests and pine plantations in the semideciduous Atlantic Forest of Argentina.

## Project description

### Study area description

This study was performed in the semideciduous Atlantic Forest of Argentina, in Misiones province. This ecosystem presents an average annual precipitation of 2000 mm, without a dry season and average temperatures of 15°C in winter (June–August) and 25°C in summer (September–March) (Ligier et al. 1990).

The native forest is composed of a complex and diverse vegetation, with three to five strata: three arboreal strata, a herbaceous stratum (50 cm high) composed of grasses and herbaceous plants and the lowest stratum dominated by mosses, saprophytes and terrestrial orchids (Morellato and Haddad 2000). The highest or emerging arboreal stratum is composed of trees up to 42 m high, usually covered with vines and epiphytes; the medium arboreal stratum reaches 30 m; and the lowest arboreal stratum or understorey consists of small trees, shrubs, woody bamboos and ferns.

Spider samples were collected from two habitats: areas of continuous native forest (Iguazú National Park, Urugua-í Provincial Park and private reserves) and areas devoted to forestry planted with the exotic conifer *Pinus taeda* (Zurita and Bellocq 2012).

## Sampling methods

### Study extent

All individuals used in this study were collected to estimate changes in the taxonomic and the functional diversity of spiders, due to the replacement of the native forest by pine plantations. Changes on taxonomic diversity were previously published in Munévar et al. 2018, whereas functional changes will be published in a future manuscript. Spiders were collected during the summer (Feb-Mar), autumn (May-Jun), winter (Jul-Aug) and spring (Sep-Oct) of 2016 in protected areas of native forest and adjacent areas of pine plantations (*Pinus taeda*); seasonal fieldwork was conducted to include the potential phenology of spider activity (Vamosi et al. 2009). Five collection methods, including pitfall traps, Winkler, entomological vacuum (G-VAC), minor and major beating; methods were used to target different vegetation strata used by spiders (ground, litter layer, herbaceous, shrubs and low arboreal stratum, respectively) (Dias et al. 2010, Azevedo et al. 2014). Minor and major beating refers to collection of spiders by shaking shrubs and low arboreal vegetation; all sampling methods related to spider collection are detailed in Munévar et al. (2018).

All the individuals collected were preserved in alcohol (80%), counted and identified to the species level or morphospecies, using taxonomic literature (i.e. Herbert and Levi 1962, Lopes-Rodrigues and Mendoça 2011, Piacentini and Grismado 2008) and by consulting with specialists from the Museo Argentino de Ciencias Naturales “Bernardino Rivadavia”, Buenos Aires, Argentina. All specimens were deposited in the spider collection of the “Instituto de Biología Subtropical (IBSI-Ara, G. Rubio) in Misiones, Argentina.

### Sampling description

Three morphological and four ecological spider traits were recorded (see below). These traits have been widely used in functional studies of arthropods due to their association with the natural history of the species and habitat use (Brousseau et al. 2018). The number of individuals measured ranged from one to five (both males and females) according to the availability of specimens collected.

**Morphological traits**

The morphological traits measured were: 1) body size, 2) femur length and 3) ocular distance (Table 1). The measurements were taken from photographs captured using a stereoscope Leica EZ4 D. All images were analyzed with Image J version 1.46r. This software allows transforming pixels to millimetres and measures distance and areas (Ferreira and Rasband 2012).

**Ecological traits**

The ecological traits were: 1) foraging strategy, 2) prey range, 3) circadian activity and 4) stratum preference (Table 1). All attributes of the traits were defined at family level using published literature (Cardoso et al. 2011, Dias et al. 2010); the presence or absence of an attribute for a determined trait is denoted by 1 or 0, respectively. Foraging strategy and prey range showed mutually exclusive attributes (e.g. spiders cannot present both, euryophagus and stenophagous diets), while stratum preference and circadian activity present multiple choice attributes (e.g. some species use both, ground and vegetation).

## Geographic coverage

### Description

The study area is located in northeast Argentina, in Misiones province. Coordinates show a polygon which encloses all sample sites (20 sites in total).

**Coordinates**: 25°48'44.72" S and 25°48'9.48" S Latitude; 54°18'58.31" O and 54°32'56.39" O Longitude.

## Taxonomic coverage

### Description

**Figure 1.**
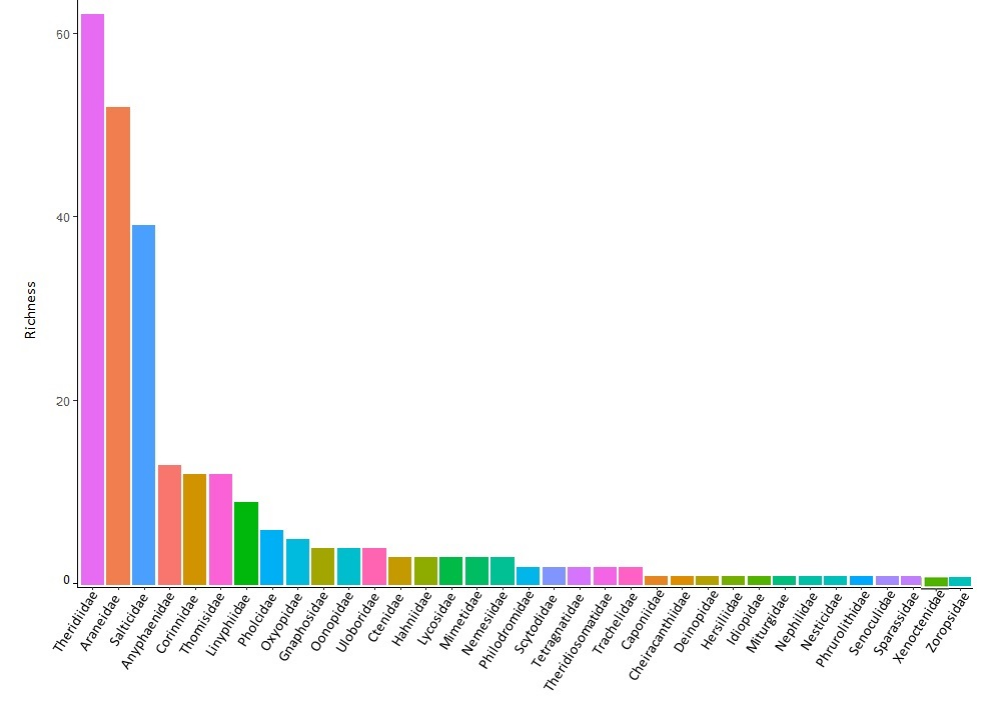
Species richness of spiders by family collected in native forests and pine plantations within the semideciduous Atlantic Forest of Argentina.

We collected a total of 15838 individuals. Only adults (32%) were identified to species/morphospecies level. We found a total of 368 species/morphospecies distributed in 38 families and 143 genera; the most species richness families were Theridiidae, Araneidae and Salticidae (62, 52 and 39 species, respectively), followed by Anyphaenidae (13 sp.), Corinnidae (12), Thomisidae (12) and Linyphiidae (9) (Fig. 1).

About 26% of the species were collected in the native forest and 40% in pine plantations; 34% of the species were shared between both sites.

From the complete assemblage, 259 species/morphospecies were selected from native forests and/or pine plantations; functional traits, previously mentioned, were assessed in 951 individuals. Species were selected, based on the availability and quality of specimens; the remaining species (109 from the total assemblage) were not in optimal conditions for measurement (in general, only one individual was captured by species).

### Taxa included

**Table:** 

Rank	Scientific Name	Common Name
order	Araneae	Spiders

## Usage rights

### Use license

Creative Commons Public Domain Waiver (CC-Zero)

## Data resources

### Data package title

Semideciduous_Atlantic_Forest_Spiders

### Number of data sets

2

### Data set 1.

#### Data set name

Traits_of_Spider

#### Data format

Tab delimited file (.csv)

#### Number of columns

10

#### Download URL

10.6084/m9.figshare.11877783

#### Description

Morphological and ecological traits of spider species in the semideciduous Atlantic Forest of Argentina.

**Data set 1.** 

Column label	Column description
Family	The full scientific name of the family in which the taxon is classified.
Scientific Name	The full scientific name.
Sex	The sex of the biological individual(s).
Body Size	Width and length of the prosoma and opisthosoma.
Femur length	Length of femur I & IV.
Ocular distance	Sum of diameters of one side eyes.
Foraging strategy	Tube web, Sheet web weaver, Space web, Orb web, Aerial hunter and Active hunter.
Prey range	Stenophagous, Euryophagous.
Circadian activity	Diurnal, Nocturnal.
Stratum preference	Ground (GR), Trunk (TR), Vegetation (VG).

### Data set 2.

#### Data set name

Ecological_data_of_spiders_communities_present_in_native_forest_and_pine_plantations.

#### Data format

Tab delimited file (.csv)

#### Number of columns

7

#### Download URL

10.6084/m9.figshare.11877777

#### Description

Dataset with presence/absence of species in each habitat type (native forests and pine plantations of different ages), number of individuals assessed, phenology (season collected) and collection method for spiders inhabiting the semi-deciduous Atlantic Forest of Argentina.

**Data set 2.** 

Column label	Column description
InstitutionCode	The name (or acronym) in use by the institution having custody of the object(s) or information referred to in the record.
CollectionID	An identifier for the collection or dataset from which the record was derived.
Scientific Name	The full scientific name, with authorship and date information, if known.
No. Individuals Measured	Number of individuals measured by species.
Presence/Absence Matrix	Presence/absence matrix of species collected in all sample sites (native forest, mature plantations, middle age plantation and young plantation).
Phenology	Seasons of spider collection: 1= winter, 2= autumn, 3= spring, 4= summer.
Collection Method	Collection methods used to capture spiders by stratum. Pitfall trap = ground, Winkler = litter layer, Entomological vacuum (G-VAC) = herbaceous stratum (0-50 cm of height), Minor beating = shrubs (50 cm-2 m of height) and Major beating = low arboreal stratum (2-6 m of height).

## Figures and Tables

**Table 1.** Traits description and features

Traits	Description	Measure	Category	Source
Body size	Body size was estimated from four measures: width and length of prosoma and, width and length of opisthosoma.	Prosoma length: Distance between anterior edge of the carapace to the posterior end in dorsal view.	Continuous (mm)	Podgaiski et al. 2013
		Prosoma width : Mayor width of caparace in dorsal view.	Continuous (mm)	Podgaiski et al. 2013
		Opisthosoma width: In the middle of the abdomen, distance from superior to inferior edge in lateral view.	Continuous (mm)	Podgaiski et al. 2013
		Opisthosoma length: Distance between apex base to posterior end of abdomen in lateral view (without spinnerets).	Continuous (mm)	Podgaiski et al. 2013
Femur length	Femur length was estimated considering legs I & IV from one side.	Femur I & IV distance from anterior edge to posterior end, in prolateral view.	Continuous (mm)	Podgaiski et al. 2013
Ocular distance	Sum of diameters of one side of the caparace eyes.	Sum of diameter of four eyes (1 ALE, 1 PLE, 1 PME, 1 AME) from one side of the caparace.	Continuous (mm)	Vandewalle et al. 2010; Nicholas et al. 2015
Foraging strategy	Foraging strategy has six attributes or levels: Tube web, Sheet web weaver, Space web, Orb web, Aerial hunter and Active hunter. All species present just one foraging strategy.	present=1; absent=0	Binary	Foelix 2011
Prey range	Diet could be euryophagus (wide food range also called polyphagous) or stenophagous (restricted food range). Both attributes are mutually exclusive.	present=1; absent=0	Binary	Pekár et al. 2011
Circadian activity	Circadian activity can be diurnal and/or nocturnal. Attributes could be multiple choices (e.g. diurnal and nocturnal).	present=1; absent=0	Multiple choices	Foelix, 2011
Stratum preference	Stratum preference could be ground (GR), trunk (TR) and/or vegetation (VG). Attributes are multiple choices.	present=1; absent=0	Multiple choices	Cardoso et al. 2011, Dias et al. 2010
